# Steps to Preventing Type 2 Diabetes: Exercise, Walk More, or Sit Less?

**DOI:** 10.3389/fendo.2012.00142

**Published:** 2012-11-19

**Authors:** Catrine Tudor-Locke, John M. Schuna

**Affiliations:** ^1^Walking Behavior Laboratory, Population Sciences, Pennington Biomedical Research CenterBaton Rouge, LA, USA

**Keywords:** walking, exercise therapy, sedentary lifestyle, training, guidelines

## Abstract

Accumulated evidence supports the promotion of structured exercise for treating prediabetes and preventing Type 2 diabetes. Unfortunately, contemporary societal changes in lifestyle behaviors (occupational, domestic, transportation, and leisure-time) have resulted in a notable widespread deficiency of non-exercise physical activity (e.g., ambulatory activity undertaken outside the context of purposeful exercise) that has been simultaneously exchanged for an excess in sedentary behaviors (e.g., desk work, labor saving devices, motor vehicle travel, and screen-based leisure-time pursuits). It is possible that the known beneficial effects of more structured forms of exercise are attenuated or otherwise undermined against this backdrop of normalized and ubiquitous slothful living. Although public health guidelines have traditionally focused on promoting a detailed exercise prescription, it is evident that the emergent need is to revise and expand the message to address this insidious and deleterious lifestyle shift. Specifically, we recommend that adults avoid averaging <5,000 steps/day and strive to average ≥7,500 steps/day, of which ≥3,000 steps (representing at least 30 min) should be taken at a cadence ≥100 steps/min. They should also practice regularly breaking up extended bouts of sitting with ambulatory activity. Simply put, we must consider advocating a whole message to “walk more, sit less, *and* exercise.”

## Introduction

Prediabetes, a state when blood glucose is elevated, yet not high enough to be classified as overt diabetes, is precursory to the development of Type 2 diabetes (Tabak et al., 2012). The American Diabetes Association (ADA) defines prediabetes as having any one of the following: (1) an impaired fasting glucose (IFG) indicated by a fasting plasma glucose (FPG) of 5.6–6.9 mmol/L without impaired glucose tolerance (IGT); (2) IGT, defined as a FPG <7 mmol/L and a 2 h oral glucose tolerance test plasma glucose concentration of 7.8–11.1 mmol/L; or (3) glycated hemoglobin A1c (HbA1c) of 5.7–6.4% (American Diabetes Association, 2011). Based on analyses of FPG or HbA1c collected from 2005 to 2008 during the National Health and Nutrition Examination Survey (NHANES), 35% of U.S. adults aged 20 years or older, and 50% of those aged 65 years or older, had prediabetes (Centers for Disease Control and Prevention (CDC), 2011). Applied to 2010 population data, this translates to an estimated 79 million prediabetic adults within the U.S. (Centers for Disease Control and Prevention (CDC), 2011). This is alarming as it is estimated that up to 70% of those with prediabetes will eventually develop Type 2 diabetes (Nathan et al., 2007). In an effort to address this public health issue, diabetes prevention programs (DPP; Eriksson and Lindgarde, 1991; Pan et al., 1997; The Diabetes Prevention Program Research Group, 1999; Uusitupa et al., 2000; Ramachandran et al., 2006) have attempted to elicit favorable changes in modifiable risk factors associated with prediabetes and Type 2 diabetes. Physical activity represents one risk factor (Hu et al., 1999, 2003b) that can be considered a logical primary target for modification in intervention research.

Physical activity recommendations for Type 2 diabetes prevention are congruent with general public health guidelines and call for a minimum of 150 min/week of moderate-to-vigorous physical activity (Physical Activity Guidelines Advisory Committee, 2008). These time-and-intensity based guidelines imply that this dose should be accumulated *above and beyond* some otherwise unquantified baseline level of non-exercise physical activity. However, it is precisely this volume of habitual behavior that has been most vulnerable to contemporary society-level energy-saving transitions evident in occupational tasks, domestic duties, transportation modes, and leisure-time preferences (Brownson et al., 2005; Church et al., 2011; Ng and Popkin, 2012). Time use studies clearly depict the dominating presence of sedentary behavior and light intensity activities during waking hours (Tudor-Locke et al., 2011b; Ng and Popkin, 2012). It is logical to forecast that public health guidelines will need future revisions to account for continued shifts toward an increasingly sedentary lifestyle (Ng and Popkin, 2012) if basal levels of non-exercise physical activity continue to erode (Hamilton et al., 2007). The looming potentially negative impact of this sedentary lifestyle shift on the prevalence of prediabetes, and ultimately Type 2 diabetes, is critical. What is needed is a clear message that conveys the total amount of exercise and non-exercise physical activity necessary to elicit important health outcomes, including reducing early risk for prediabetes and ultimately Type 2 diabetes. This article will discuss the potential for an expanded message which not only emphasizes exercise, but also stresses the importance of more non-exercise physical activity (e.g., walking) and less sedentary time (e.g., sitting) as a complete public health strategy for improving the metabolic profile of individuals with prediabetes. To begin, we clarify terms used repeatedly herein.

## Physical Activity vs. Energy Expenditure

The term “physical activity” was defined by Caspersen et al. (1985) as “any bodily movement produced by the skeletal muscles that results in energy expenditure.” This definition intentionally captured the broadest spectrum of human movement unrestricted in terms of duration, intensity, or frequency. In contrast, “energy expenditure,” the metabolic cost of living, is shaped by sex, age, and body mass, and includes contributions from resting metabolic rate, the thermic effect of feeding, and the metabolic costs of movement (Lamonte and Ainsworth, 2001).

Day-to-day variability in energy expenditure within individuals is primarily the result of differences in physical activity behaviors (Westerterp, 2008), whereas between-individual variability in energy expenditure is largely influenced by differences in body mass (Masse et al., 2004). Since energy expenditure is related to, but not synonymous with physical activity, measures of energy expenditure can only be considered *indirect* estimates of physical activity (i.e., movement) across individuals, even with adjustments for body mass (Masse et al., 2004). Within individuals, lowered energy expenditure following short-term reductions in physical activity promotes a positive energy balance and subsequent increases in intra-abdominal fat (Olsen et al., 2008). However, the potentially deleterious effects of reduced physical activity on the body’s metabolic processes likely extend beyond the intrinsic obesity-generating demerits of compromised energy balance (Hamilton et al., 2007).

## Exercise

Exercise has been defined as a subset of physical activity that “is planned, structured, and purposive in the sense that improvement or maintenance of one or more components of physical fitness is an objective” (Caspersen et al., 1985). The American College of Sports Medicine (ACSM) published a series of position stands describing the recommended quantity (i.e., frequency in days/week, and duration in min/day and min/week) and quality (i.e., intensity in metabolic equivalents or METs; 1 MET being equivalent to 3.5 mL of O_2_ consumption per kg body weight per minute) of *exercise* necessary to develop and maintain fitness in healthy adults (American College of Sports Medicine, 1978, 1990, 1998; Garber et al., 2011). In regard to cardiorespiratory training, the most recent position stand states: “The ACSM recommends that most adults engage in moderate-intensity cardiorespiratory *exercise training* for ≥30 min/day on ≥5 days/week for a total of ≥150 min/week, vigorous-intensity cardiorespiratory exercise training for ≥20 min/day on ≥3 days/week (≥75 min/week), or a combination of moderate- and vigorous-intensity exercise to achieve a total energy expenditure of ≥500–1000 MET/min/week.” This recommendation is congruent with the first U.S. *Physical Activity* Guidelines for Americans (Physical Activity Guidelines Advisory Committee, 2008) published in 2008: “For substantial health benefits, adults should do at least 150 min (2 h and 30 min) a week of moderate-intensity, or 75 min (1 h and 15 min) a week of vigorous-intensity aerobic physical activity, or an equivalent combination of moderate- and vigorous-intensity aerobic activity. Aerobic activity should be performed in episodes of at least 10 min, and preferably, it should be spread throughout the week.”

Chronic moderate and vigorous aerobic exercise (6–12 months) reduces insulin resistance (Houmard et al., 2004; Evans et al., 2005; Bajpeyi et al., 2009). However, evidence suggests that the duration of exercise is more important than intensity for eliciting favorable responses in insulin action (Houmard et al., 2004). Regular training enhances skeletal muscles’ responsiveness to insulin by increasing activity and/or expression of proteins involved in insulin signaling and glucose metabolism (Christ-Roberts et al., 2004; O’Gorman et al., 2006; Wang et al., 2009). Fat oxidation also plays a role in improved insulin action and aerobic exercise increases skeletal muscles’ lipid storage and fat oxidation capacity (Duncan et al., 2003; Goodpaster et al., 2003; Pruchnic et al., 2004; Kelley and Kelley, 2007).

**Figure 1:**
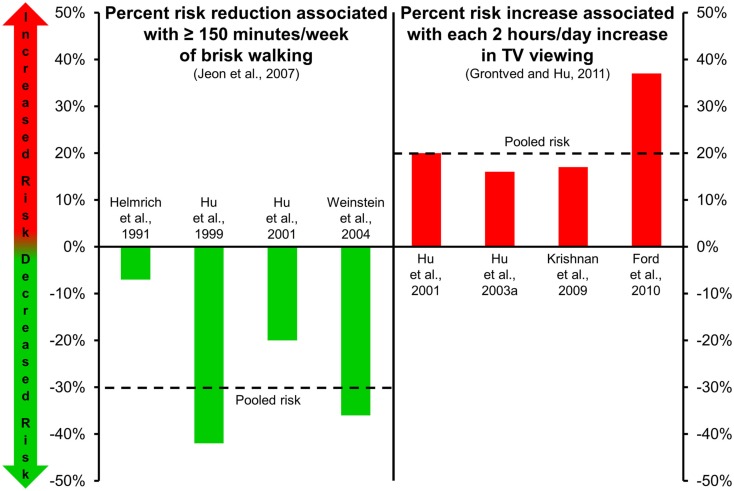
**Risk of type 2 diabetes associated with walking (left panel; Jeon et al., 2007) and TV viewing (right panel; Grontved and Hu, 2011)**. Dashed lines in each panel represent the pooled risk among the studies included. Risk reduction associated with ≥150 min/week of brisk walking is in comparison to risk level associated with minimal amounts of weekly walking.

In addition to the favorable metabolic effects associated with regular exercise, epidemiological data from large prospective cohort studies indicate that moderate exercise, such as walking, and more vigorous activities, protects against the development of Type 2 diabetes (Helmrich et al., 1991; Manson et al., 1991; Hu et al., 1999). A synthesis of results from four cohort studies (Helmrich et al., 1991; Hu et al., 1999, 2001; Weinstein et al., 2004) indicates that brisk walking for at least 150 min/week, when compared to minimal amounts of weekly walking, significantly lowers risk for Type 2 diabetes [relative risk = 0.70 (95% CI 0.58–0.84); Figure 1, left panel; Jeon et al., 2007]. Moreover, evidence suggests that incrementally higher amounts of exercise provide greater protective benefits against Type 2 diabetes (Hu et al., 1999).

Among individuals already at risk for Type 2 diabetes, several randomized-controlled trials provide further evidence supporting the beneficial role of exercise (Pan et al., 1997; Tuomilehto et al., 2001; Knowler et al., 2002). As an example, the U.S. DPP reported that diabetes incidence after nearly 3 years of follow-up was 58% lower among participants at risk for Type 2 diabetes enrolled in a “lifestyle” intervention group (including weight loss, diet, and moderate-intensity exercise for at least 150 min/week) than those enrolled in a control group, and 39% lower than those enrolled in a “drug” treatment group (prescribed metformin; Knowler et al., 2002). Unfortunately, objectively monitored data indicates that <10% of the U.S. adult population exercises at this amount and intensity (Troiano et al., 2008; Tucker et al., 2011). However, associations between sedentary behavior and mortality from all causes and cardiovascular disease are independent of exercise (i.e., leisure-time physical activity; Katzmarzyk et al., 2009) and suggest that a “deficiency of non-exercise physical activity” (Hamilton et al., 2007) exchanged for excesses in sedentary behavior may diminish the beneficial effects of exercise. This threat should not be any different for people with prediabetes.

## Non-Exercise Physical Activity

If “physical activity” represents the full spectrum of human movement, and “exercise” is a subcategory representing the higher end of this spectrum (Caspersen et al., 1985), then the remainder can be conceptualized as “non-exercise physical activity.” Of all types of physical activity, public health and clinical practice efforts most commonly encourage walking (Siegel et al., 1995). “Walking for exercise” is the most commonly reported form of exercise (Ham et al., 2009). But other forms of daily walking, including shopping, touring, and walking the dog (Tudor-Locke and Ham, 2008), to name but a few examples of other forms of walking behaviors, can more appropriately be classified as “non-exercise physical activity.” Specific to incident diabetes, a prospective study of 37,828 women from the Women’s Health Study found that self-reported walking for 2–3 h/week was associated with a 34% reduction in the incidence of Type 2 diabetes over almost 7 years of follow-up (Weinstein et al., 2004). Objectively measured walking (higher steps/day) has also been associated with lower levels of fasting glucose (Strath et al., 2007) and with reduced 5-year risk of incident dysglycemia (the effect was not fully mediated through reduced adiposity; Ponsonby et al., 2011). Interrupting extended bouts of sitting with light or moderate-intensity walking improves postprandial glucose and insulin levels in overweight/obese adults (Dunstan et al., 2012).

Unfortunately, there is mounting evidence that walking has eroded from our day-to-day lifestyle (Ng and Popkin, 2012). As an example, traditional Amish women and men take 14,000–18,000 steps/day (Bassett et al., 2004) whereas recent U.S. estimates indicate typical modern adults take 5,900–6,900 steps/day (Tudor-Locke et al., 2004, 2009; Wyatt et al., 2005). Evidence is also emerging to suggest that the metabolic ramifications of this behavioral transition are not trivial. Mikus et al. (2012) studied healthy, active individuals who transitioned from averaging >10,000 steps/day to <5,000 steps/day for only 3 days to determine if this abrupt and temporary change in daily physical activity would modify postprandial and overall glycemic control as determined by continuous glucose monitors. Not surprisingly, the >5,000 steps/day reduction in ambulatory activity produced a 2.5 h/day increase in sitting time. After only 3 days of reduced ambulatory activity and increased sitting, significant increases in average glucose excursions were observed following meals.

## Sedentary Behaviors

Sedentary behaviors have been defined as those activities with a relatively low rate of energy expenditure (1–1.5 METs; Owen et al., 2000; Pate et al., 2008), a relative lack of movement as detected by objective monitoring devices (Matthews et al., 2008; Wong et al., 2011), and/or sitting postures (Kozey-Keadle et al., 2011; Owen et al., 2011). Even people who exercise may accumulate large amounts of sedentary behavior (Tremblay et al., 2010), and therefore exercise and sedentary behaviors are considered to be independent of each other (Tremblay et al., 2010). In contrast, sedentary behaviors appear to have systematically replaced non-exercise physical activity (Ng and Popkin, 2012); there is an almost complete inverse association (*r* = −0.96) between these two behaviors (Healy et al., 2007, 2008b; Tremblay et al., 2010). The current body of literature examining metabolic responses to bouts of sedentary behavior is limited. However, several studies have reported increases in insulin (up to 67%) during a 2 h oral glucose tolerance test following extended periods of bed rest (Blanc et al., 2000; Hamburg et al., 2007). Plasma glucose levels may also significantly rise following extended periods of bed rest (Zorbas et al., 1999). Since bed rest studies represent an extreme case of enforced reclining/lying behavior, the generalizability of these findings to more common lifestyle-based patterns of prolonged sitting interspersed with natural movement can be questioned. However, recent research has shown that just a single day of prolonged sitting reduces insulin action (Stephens et al., 2011), and, as mentioned above, breaking up extended sitting time with short bouts of light or moderate-intensity walking may help moderate such effects and lower postprandial glucose and insulin levels (Dunstan et al., 2012).

Results from several large epidemiological studies indicate that higher amounts of sedentary time are associated with greater risks for obesity (Hu et al., 2003a), the metabolic syndrome (Ford et al., 2005; Sisson et al., 2009), and Type 2 diabetes (Hu et al., 2003a). Specifically, higher amounts of self-reported leisure-time sedentary behavior (e.g., screen-based behaviors including computer gaming, TV watching, etc.), and also usual sitting in the context of daily occupational/domestic work, is associated with a higher odds of the metabolic syndrome and individual cardiovascular disease risk factors (including elevated glucose; Sisson et al., 2009). Moreover, objectively measured sedentary time has been associated with clustered metabolic risk independent of time spent in moderate-to-vigorous physical activity (Healy et al., 2008b). In particular, TV viewing is one of the most frequently reported sedentary behaviors (second only to eating and drinking) as captured in time use studies (Tudor-Locke et al., 2010). A meta-analysis (Grontved and Hu, 2011) of four studies (Hu et al., 2001, 2003a; Krishnan et al., 2009; Ford et al., 2010) evaluating relationships between TV viewing and risk of Type 2 diabetes (175,938 individuals, 6428 incident cases during 1.1 million person-years of follow-up) reported that pooled relative risks were 1.20 per every 2 h/day of TV viewing (Figure 1, right panel). The estimated absolute risk difference per every 2 h of TV viewing per day was 176 cases of Type 2 diabetes per 100,000 individuals per year.

## The Whole Message: Walk More, Sit Less, and Exercise

It is apparent that a movement toward a “whole message” has been slowly evolving. As noted above, U.S. public health guidelines focus on promoting moderate-to-vigorous-intensity exercise, however, recent iterations do indeed acknowledge “more (activity) is better than none,” especially in those who are considered inactive (Physical Activity Guidelines Advisory Committee, 2008). The ACSM included a steps/day recommendation in their recent update on the quantity and quality of exercise necessary to improve cardiorespiratory health outcomes (Garber et al., 2011). Finally, Canadian scientists have been the first to issue public health recommendations in regard to reducing sedentary behaviors (e.g., sitting), and replacing them with movement (most obviously walking behaviors; Tremblay et al., 2011, 2012). These messages appear to be also relevant to treating prediabetes and preventing Type 2 diabetes.

**Figure 2:**
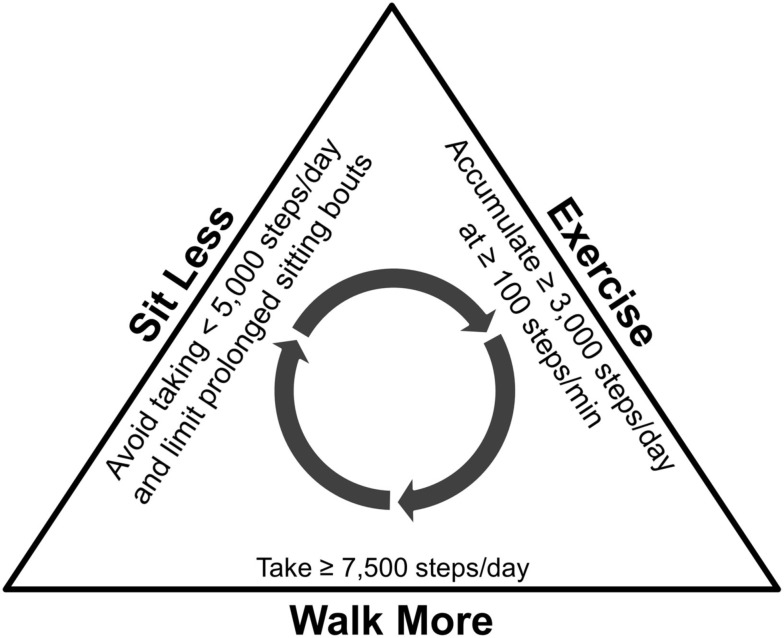
**Graphic representation of the message to “walk more, sit less, *and* exercise”**.

The whole message can be captured by advocating that adults “walk more, sit less, *and* exercise.” Although the simplicity of this message is appealing, its lack of explicitly measureable parameters conveys only vague encouragement that cannot be easily tracked or otherwise evaluated. This is challenging, since as some may argue, at least in terms of walking behaviors, there maybe no apparent threshold effect: more is better, wherever your base is. Still, “walking more” can be broken down into daily frequency and accumulating greater amounts of time at higher cadences (steps/min; Tudor-Locke, in press). Specifically, we recommend that adults avoid days of taking <5,000 steps/day (Tudor-Locke et al., in press) and strive to average ≥7,500 steps/day (Tudor-Locke et al., 2011a,c), of which ≥3,000 steps (representing at least 30 min; Tudor-Locke et al., 2005; Marshall et al., 2009) should be taken at a cadence ≥100 steps/min (Tudor-Locke et al., 2005; Marshall et al., 2009; Beets et al., 2010; Abel et al., 2011; Rowe et al., 2011). They should also be encouraged to regularly break up extended bouts of sitting with ambulatory activity (Healy et al., 2008a; Dunstan et al., 2012). Reducing/interrupting sedentary behavior is compatible with “walking more” (Healy et al., 2007, 2008b; Tremblay et al., 2010) so a simple accounting of daily steps could be used to indirectly track sedentary behavior (Tudor-Locke et al., in press). A viable target sedentary behavior to limit/interrupt is prolonged television watching (i.e., >2 h/day; Sisson et al., 2009; Tudor-Locke et al., 2010; Grontved and Hu, 2011). However, since sedentary behaviors are ubiquitous in daily living (Matthews et al., 2008), there are ample opportunities to sit less, or perhaps more clearly, intermittently replace sitting by interjecting more bouts of walking throughout the day (Dunstan et al., 2012). Figure 2 presents a graphic representation of this message to “walk more, sit less, *and* exercise.”

## Summary

In summary, prediabetes is on the rise, and with it comes the looming specter of Type 2 diabetes. Exercise is a recognized potent strategy for treating prediabetes and preventing Type 2 diabetes. Contemporary societal changes in lifestyle behaviors have resulted in a notable widespread deficiency of non-exercise physical activity exchanged for an excess in sedentary behaviors. It is possible that the known beneficial effects of exercise are undermined against this backdrop of normalized slothful living. Public health guidelines have historically focused on an exercise message, but it is evident that the need to revise the message to reflect this lifestyle shift is necessary. Simply put, we must consider advocating a whole message to “walk more, sit less, and exercise.”

## Conflict of Interest Statement

The authors declare that the research was conducted in the absence of any commercial or financial relationships that could be construed as a potential conflict of interest.
